# *Extreme Physiology & Medicine*: a new journal focussed on integrative human physiology under stress

**DOI:** 10.1186/2046-7648-1-1

**Published:** 2012-09-04

**Authors:** Michael PW Grocott, Hugh E Montgomery

**Affiliations:** 1 Integrative Physiology and Critical Illness Group, Division of Clinical and Experimental Science, Faculty of Medicine, University of Southampton, University Road, Southampton, SO17 1BJ, UK; 2 Anaesthesia and Critical Care Research Unit, University Hospital Southampton, NHS Foundation Trust, CE.93 Mailpoint 24, E-Level Centre Block, Tremona Road, Southampton, SO16 6YD, UK; 3 The Royal College of Anaesthetists, Churchill House 35 Red Lion Square, London, WC1R 4SG, UK; 4 UCL Institute of Human Health and Performance, University College London, 5 University Street, London, WC1E 6JF, UK; 5 Critical Care Medicine, The Whittington Hospital NHS Foundation Trust, Magdala Avenue, London, N19 5N, UK

## 

Welcome to the launch issue of *Extreme Physiology & Medicine* - a new open-access journal focussing on the integrative physiology of environmental, exertional and clinical stress in humans [1].

The study of integrated human physiology underpins our understanding of human pathophysiology. The characterisation of responses to external stressors - whether relating to changes in hydration, humidity or hypoxia, exertion or altitude, barometric pressure or gravity - has been especially informative. Importantly, understanding of disease states such as critical illness (in which similar challenges to oxygen delivery, thermoregulation and hydration exist) can be informed through such study [2,3]. However, recent decades have seen a waning of investment in integrative physiology in general and environmental physiology in particular [4]. Just as they have in the world of global politics and finance, revolutions in the fields of technology and computation, genomics and molecular biology, have dominated the biomedical sphere. Whilst this has been captivating and exciting, we are now learning that not all human physiology or pathophysiology can be ‘e-explored’ or understood through reductionist molecular biology alone. Whilst such studies can be hugely enhanced through the use of new technologies, there remains much to learn from the integrative physiological study of ‘whole humans’ [5].

But where do the observations derived from such experiments find a home? Whilst the field of exercise physiology is reasonably served, there are few journals which publish data specifically relating to ‘extreme’ or ‘boundary’ physiology, or serve to encourage translation from these findings to the clinical setting. Fewer still encourage reflection and speculation in these spheres.

*Extreme Physiology & Medicine* hopes to address these deficits by providing a rapid, receptive and reliable route for the publication of relevant high-quality articles. Articles will be peer reviewed by recognised world leaders in their fields, ensuring the highest standard of quality control. The ‘open access’ model of publishing brings with it distinct advantages in terms of speed of publication and global visibility (to all who are connected to the internet) [6-8]. It is the great democratic force within biomedical research publication. Authors retain copyright and grant anyone the right to reproduce and disseminate their article. Open access facilitates more downloads, citations and impact [9-11]. There is no need for radical restriction of content: space for the presentation of data, tables and even video can be (quite literally) limitless. Reference datasets can be co-posted, to encourage free exchange of views and analyses. However, all these do come with a small price: the substitution of subscription charges with a fee payable by the authors (or their submitting institution). Increasingly, such fees are considered ‘the last phase of the funded research process’, funded by grant bodies and institutions [12-15].

So what will *Extreme Physiology & Medicine* publish? Our focus will be on integrative human physiology under conditions of physiological stress, be it environmental, exertional or clinical. Underpinning the journal is the concept that the study of human systems under extreme stress can enhance understanding of disease processes and the treatment of patients; we particularly welcome papers that recognise the relevance of boundary physiology to human disease states and their management. What can be learned from weightlessness that can be applied to the frail facing bed rest (or vice versa)? What can we learn from studies of anabolic training that can be applied to those with disease-associated muscle wasting? Can we learn anything from bone remodelling in exercise that is of relevance to bone weakness in osteoporosis or from mountaineers that might help those facing limited oxygen availability at sea level? *In vitro* and animal studies will be considered - but only where they represent a frame-shift in understanding, or an important methodological contribution to, human physiological research.

We have tried to make space for the once-popular (and, in our view, important and undervalued) opportunity to report a novel observation (even if it is hard to explain). We hope to provide a forum where authors can reflect on what we do not know as well as what we (think we) do. In addition to research reports (which can be long or short), we will encourage scholarly commentary and review as well as presentation of case reports and experimental observations. Manuscripts reporting the methods of significant studies will be welcomed. We look forward to receiving and publishing new hypotheses and to publishing the reflections of some of the world's greatest physiologists. We aim to produce a rigorous, stimulating, and highly regarded ‘top-tier journal’. However, this will only happen if you want it to be so. In this regard, we are grateful to our editorial board of international leaders in their field and to you as the readers of and contributors to *Extreme Physiology & Medicine*.

These first articles published in *Extreme Physiology & Medicine* showcase our mission to look across the range of integrative physiology under stress from around the globe. They include original articles describing environmental conditions high on Mount Everest and the impact of this environmental stress on mountaineers [16], an investigation of skeletal muscle volume following dehydration induced by exercise in heat [17] and a fascinating case report describing a recreation of Priestley's classic bell jar experiment with a human subject [18].

We hope you enjoy *Extreme Physiology & Medicine* and look forward to your comment and submissions.

## Authors' information

MPWG is the professor of Anaesthesia and Critical Care Medicine and head of the Integrative Physiology and Critical Illness Group, Division of Clinical and Experimental Science, Faculty of Medicine, University of Southampton. He is also a consultant in critical care medicine at University Hospital Southampton NHS Foundation Trust and the British Oxygen Comany Professor of Anaesthesia. HEM is the Director of the UCL Institute for Human Health and Performance and Professor of Intensive Care Medicine, University College London. He is also a consultant intensivist, Whittington Hospital NHS Trust.
